# Toxic effects of decabromodiphenyl ether (BDE-209) on human embryonic kidney cells

**DOI:** 10.3389/fgene.2014.00118

**Published:** 2014-05-06

**Authors:** Min Li, Zhenping Liu, Liang Gu, Rong Yin, Huarong Li, Xiaobai Zhang, Tongcheng Cao, Cizhong Jiang

**Affiliations:** ^1^Shanghai Tenth People's Hospital, Shanghai Key Laboratory of Signaling and Disease Research, The School of Life Sciences and Technology, Tongji UniversityShanghai, China; ^2^Department of Chemistry, Tongji UniversityShanghai, China; ^3^Department of Otorhinolaryngology, Jianyang Municipal HospitalJianyang, China

**Keywords:** PBDE, BDE-209, HEK293T, gene expression, toxicity, nucleosome, carcinogenicity

## Abstract

Polybrominated diphenyl ethers (PBDEs) are widely used as flame-retardant additives in consumer and household products and can escape into the environment over time. PBDEs have become a global environmental organic pollutant due to the properties of persistence, toxicity, and bioaccumulation. The well-studied toxic effects of PBDEs mainly include thyroid hormone disruption and neurotoxicity. There is no consistent conclusions on the carcinogenic potential of PBDEs to date. Here, we explored the toxic effects of BDE-209 on human embryonic kidney cells (HEK293T). The comparison of the gene expression profiles of HEK293T cells with BDE-209 treatment and the negative control found that BDE-209 exposure may alter nucleosome organization through significantly changing the expression of histone gene clusters. The remodeled chromatin structure could further disturb systemic lupus erythematosus as one of the toxic effects of BDE-209. Additionally, gene sets of different cancer modules are positively correlated with BDE-209 exposure. This suggests that BDE-209 has carcinogenic potential for a variety of tumors. Collectively, BDE-209 has a broader toxicity not limited to disruption of thyroid hormone-related biological processes. Notably, the toxic effects of BDE-209 dissolved in dimethyl sulfoxide (DMSO) is not the simply additive effects of BDE-209 and DMSO alone.

## Introduction

Polybrominated diphenyl ethers (PBDEs) are a major type of brominated flame retardants. PBDEs have been widely used in a variety of consumer and household products to delay the ignition of materials (Birnbaum et al., 2003; Hale et al., 2003). The previous study reported that PBDEs in the household goods could leach into the environment (Kim et al., 2006). It has been reported that PBDEs were detected in house dust samples (Stapleton et al., 2005). Because of its persistence, toxicity, and bioaccumulative nature, PBDEs have become global environmental contaminants (Costa and Giordano, 2007; Tseng et al., 2008). PBDE residues have been detected in serum, breast milk, and adipose tissue from individuals (Birnbaum et al., 2003; McDonald, 2005). Moreover, concentrations of PBDEs in human and animal bodies are increasing (McDonald, 2002).

The toxic effects of PBDEs can lead to thyroid hormone disruption, neurobehavioral toxicity, and cancer. The chemical structure of PBDEs is very similar to that of the thyroid hormones (TH), 3,3′,5-triiodothyronine (T3) and 3,3′,5,5′-tetraiodothyronine (T4). The metabolites of PBDEs compete with thyroid hormones to bind to thyroid hormone receptors (TR) (Marsh et al., 1998)and thyroid hormone transport proteins (Meerts et al., 2000). As a consequence, PBDEs disrupt TH homeostasis. The T4 level was decreased in rats and mice following oral administration of PBDEs (Fowles et al., 1994; Darnerud and Sinjari, 1996). Another possible mechanism of thyroid hormone disruption is that PBDEs could induce different families of liver enzymes (Von Meyerinck et al., 1990; Fowles et al., 1994; Zhou et al., 2001). Thus, PBDEs may alter many signal transduction pathways regaled by TH at multiple levels. For example, PBDEs significantly suppressed Purkinje cell dendrite arborization by disrupting TR-mediated transcription (Ibhazehiebo et al., 2011).

Studies showed that PBDE exposure exerted neurotoxicity. TH regulates the development of the nervous system at multiple levels, including proliferation of neuronal and glial cells, cytoskeletal assembly and stability essential for migration and neuronal outgrowth (Porterfield, 2000). Therefore, the normal TH levels are critical for proper brain development (Porterfield and Hendrich, 1993; Morreale De Escobar et al., 2000). Transthyretin, the TH transport protein, functions in delivering TH to the fetus from the mother during gestation (Brouwer et al., 1998). As introduced above, TH could compete with TH for transthyretin and cause TH imbalance that could adversely affect brain development. A study of neurobehavioral effects of PBDEs found that abnormal TH level in the mother usually results in reduced intelligence in progeny (Morreale De Escobar et al., 2000). More studies showed that newborns exposed in PBDEs developed learning and motor deficits as they grew older (Eriksson et al., 1998, 1999; McDonald, 2002).

There is evidence indicating the carcinogenic potential of the fully brominated deca-BDE (NTP, 1986). The national toxicology program (NTP) found clear correlation between PBDE dose increase and liver neoplastic nodules in rats. The program study also showed the increased acinar cell adenoma of the pancreas in male rats treated with high-dose PBDEs. In contrast, only marginal increases in liver and thyroid tumors were observed to be related to deca-BDE. Interestingly, another earlier bioassay in rats with much lower dose of deca-BDE did not found statistically significant increases in tumors (Kociba et al., 1975). The lower molecular weight PBDEs (tri- to hexa-BDEs) are more bioaccumulative than deca-BDE. Unfortunately, there is only limited data regarding carcinogenicity of these more bioaccumulative PBDEs. Therefore, more studies should be undertaken to understand the carcinogenic potential of PBDEs.

In this study, we employed the high-throughput technology RNA-seq to profile the global gene expression of human embryonic kidney cells (HEK293T) cultured in the normal medium, and the medium added with DMSO (the solvent) and with BDE-209 dissolved in DMSO. Pairwise comparisons identified significantly expressed genes between each two samples. The functional analysis of these genes found that BDE-209 exposure may alter nucleosome arrangement through changing the expression of histone gene clusters. In addition to this, BDE-209 exposure could increase the risk for tumorigenesis of a number of tumors. Our results also found that the total toxic effects of BDE-209 dissolved in DMSO led to a more complex toxic effects than the additive effects of BDE-209 and DMSO alone.

## Materials and methods

### Cell culture and BDE-209 treatment

We cultured human embryonic kidney 293 cells (HEK293T) at 37°C in 5% CO_2_ as adherent monolayer in Dulbecco modified Eagle medium (DMEM) (Hyclone) that was supplemented with L-glutamine and 10% fetal bovine serum (FBS) (Hyclone). BDE-209 was dissolved in the dimethyl sulfoxide (DMSO) and added to the culture medium. The final concentration of BDE-209 and DMSO is 10^−6^ and 10^−3^ M, respectively. The negative control is the HEK293T cells cultured in the normal medium. The DMSO treatment sample is the HEK293T cells cultured in the medium added with DMSO. The BDE-209 treatment sample is the HEK293T cells cultured in the medium added with BDE-209 dissolved in DMSO. All samples were cultured for 48 h.

### RNA-seq experiment

We extracted the total RNA w from each sample using Trizol reagent (Invitrogen) according to the manufacturer's instructions. Then, BGI-Shenzhen (the sequencing service provider) enriched the mRNA by polyA tail, prepared the library, and did the sequencing using Illumina HiSeq2000. Sequencing method was 49 bp single-end reads. RNA-seq data can be accessed through GEO database under accession number GSE56361.

### Bioinformatics analysis

RNA-seq reads were aligned to the hg19 genome with SOAPaligner-v2.21 (Li et al., 2009), allowing maximal two mismatches per read. Only the uniquely mapped reads were retained for further analysis in this study. Gene and genome coverage analysis were calculated by SOAPcoverage. For each transcript, Reads Per Kilobase per Million mapped reads (RPKM) were calculated to evaluate the expression level. The gene expression change was estimated using NOISeq (Tarazona et al., 2011). Genes with two or more fold expression change and NOISeq *q*-value ≥ 0.6 were defined as the differentially expressed (DE) genes. Gene ontology (GO) and pathway analysis for DE genes were performed using the tool DAVID (Huang Da et al., 2009a,b). Gene Set Enrichment Analysis (Mootha et al., 2003; Subramanian et al., 2005) was performed using the gene sets of oncogenic signature (C6) and computational gene sets (C4)—cancer modules from cancer-oriented microarray data, BDE-209 or DMSO treatment as the phenotype data, the expression profiles as the data set. Metric for ranking genes was set to Diff_of_classes. Other parameters used default values. All the gene sets were curated by MSigDB database v4.0.

## Results

### Gene expression profiling of human embryonic kidney cell HEK293T with BDE-209 treatment

**Figure 1:**
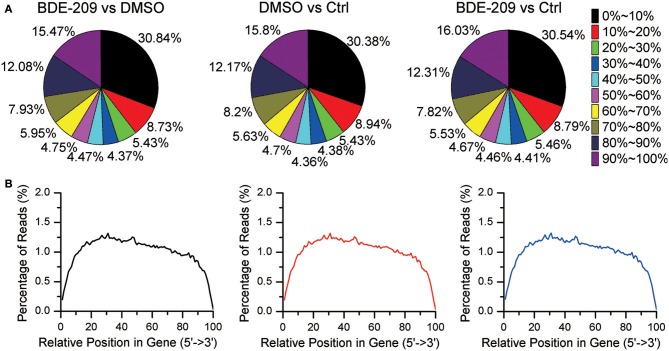
**Gene coverage and RNA integrity of RNA sequencing data. (A)** Gene coverage is the proportion of a gene region covered by RNA-seq reads and binned with an interval of 10%. Each gene belongs to one of the bin. **(B)** Distribution of RNA-seq reads along gene bodies.

In order to explore the effects of PBDEs on gene expression in HEK293T cell, we employed RNA-seq technology to obtain the global gene expression profiles of HEK293T cell treated with BDE-209 of environmental relevant concentration (10^−6^ M). We also generated transcriptomic profiles of HEK293T cell treated with the solvent DMSO (10^−3^ M) and normal medium as negative control, respectively. Pairwise comparison of the gene expression profiles of the three samples will allow us not only to analyze the impacts of BDE-209 and DMSO alone on gene expression but also the additive effects of two organic chemicals. More than 86% of reads were mapped to the reference genome (Table S1). The percentage of N (ambiguous nucleotide) at each read position is extremely low (Figure S1). RNA integrity examination showed no obvious degradation. Moreover, the sequencing reads reach a high gene coverage such that more than 60% of the annotated genes have a coverage rate higher than 60% (Figure 1). Taken together, our sequencing data well represent expressed genes and reflect the expression level of genes.

### BDE-209 exposure leading to perturbation in nucleosome organization

**Figure 2:**
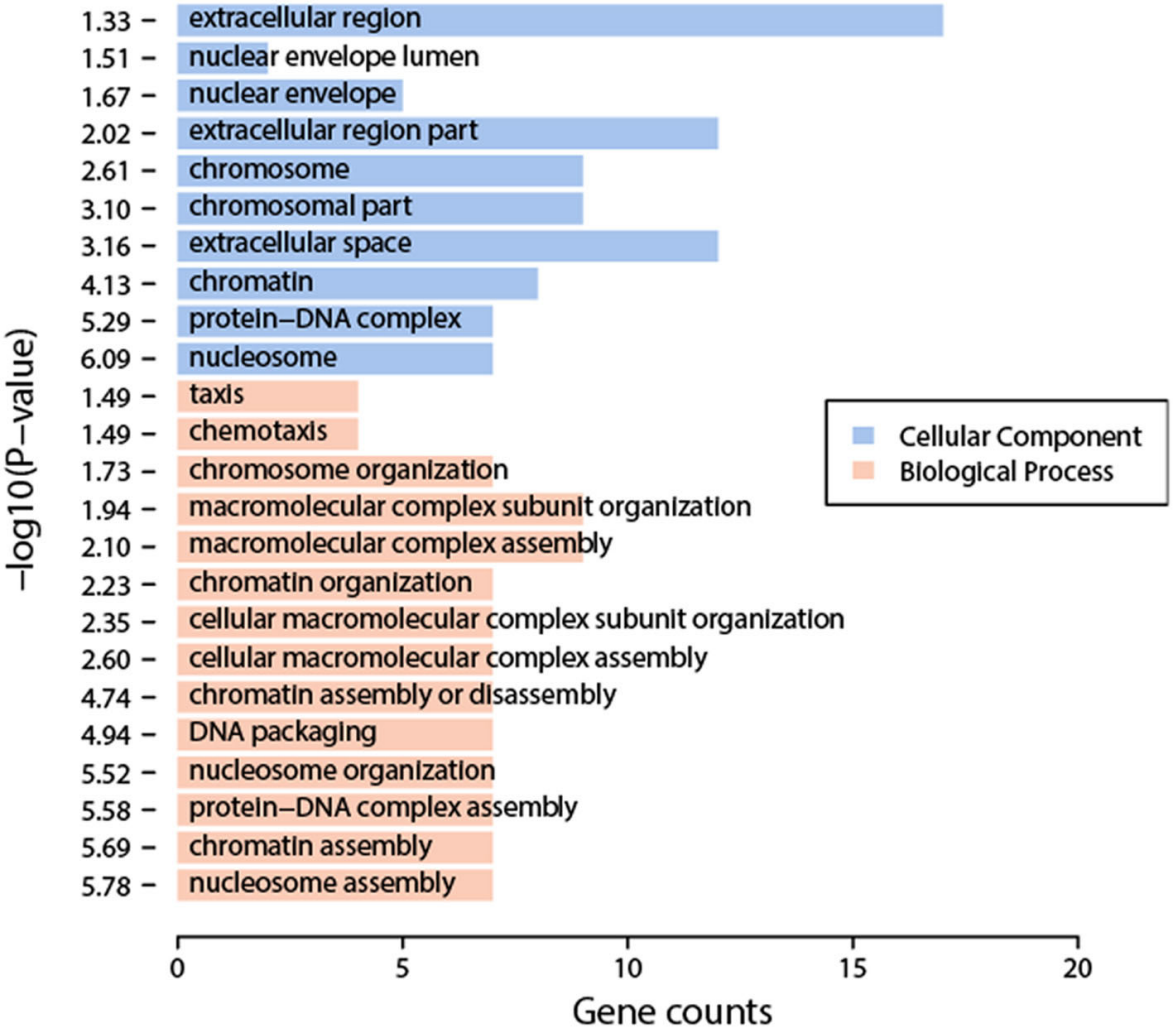
**Enriched GO terms of differentially expressed genes between BDE-209 and DMSO samples**. Biological process GO terms (pink bars) are mainly related to chromatin remodeling such as nucleosome organization, nucleosome assembly, DNA packaging, etc. Cellular component GO terms (blue bars) are also largely located in chromosome, nucleosome, etc.

We compared the transcriptomic profiles between BDE and the solvent DMSO samples, and identified 94 statistically significantly differentially expressed genes, 62 up-regulated genes and 32 down-regulated genes (Table S2). The functional analysis of these genes found that they were enriched in nucleosome organization related biological process GO terms, including chromosome organization, nucleosome organization, DNA packaging, etc. Interestingly, these genes are also enriched in chromatin or nucleosome related cellular component GO terms, including chromosome part, nucleosome, etc. (Figure 2). The impacts on nucleosome organization could be largely due to the gene clusters encoding histones that were differentially expressed in the BDE-209 treatment (Table S2). Nucleosome occupancy controls the accessibility of DNA sequences and regulates the binding of transcription factors to their target sites. Thus, nucleosome positioning affects a variety of biological processes including DNA duplication, DNA repair, transcription, and more. This indicates that BDE-209 is likely an epigenetic factor regulating gene expression through nucleosome remodeling. Further analysis found that the differentially expressed genes were enriched in the pathway systemic lupus erythematosus (*p*-value = 1e-5.32). The KEGG pathway annotation shows that nucleosome and histone gene clusters are the upstream factors in the pathway systemic lupus erythematosus. Therefore, the perturbation of BDE-209 to systemic lupus erythematosus is likely attributed to the differentially expressed gene clusters encoding histones in the BDE-209 treatment (Table S2).

### Effects of BDE-209 dissolved in DMSO on gene expression is not additive effects of BDE-209 and DMSO

**Figure 3:**
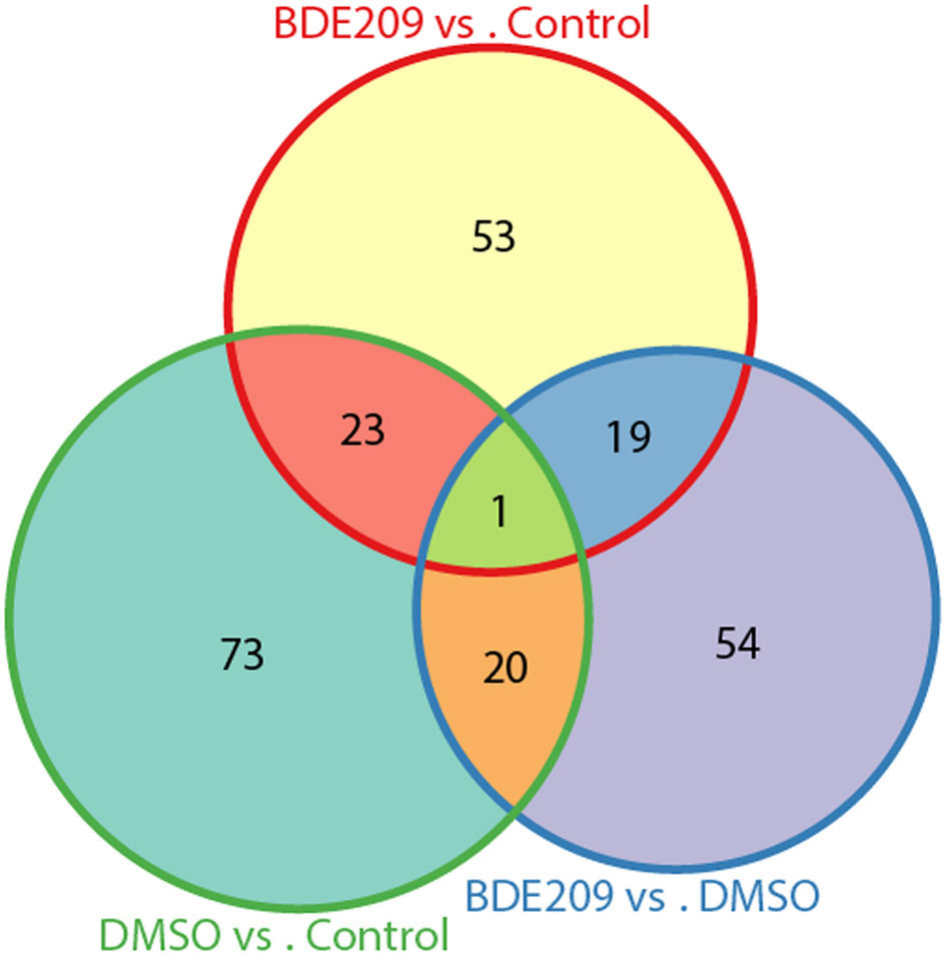
**Only small portion of genes are common among the three lists of differentially expressed genes from pairwise comparisons of the three samples**. The three lists of genes have only one gene in common. There are only about 20 common genes (~25%) shared by each two of the gene sets, respectively.

DMSO is a colorless and odorless organosulfur compound that is an important organic solvent. It is also a toxic chemical. Comparison of the gene expression profiles between DMSO and the negative control (normal medium only) samples identified 117 significantly differentially expressed genes (defined gene Set I hereinafter), 72 up-regulated genes and 45 down-regulated genes. Similarly, we obtained 96 significantly differentially expressed genes (defined gene Set II hereinafter) between BDE-209 dissolved in DMSO and the negative control samples, 54 up-regulated genes and 42 down-regulated genes. We defined the above identified 94 significantly differentially expressed genes between BDE-209 dissolved in DMSO and DMSO samples as gene Set III. There are only about 20 common genes (~25%) shared by each two of the gene sets, respectively (Figure 3). Gene Set III should represent the effect of BDE-209 alone on gene expression in principle. Gene Set II should reflect the combined effect of BDE-209 and DMSO. However, less than half genes of Set II overlapped with the union of gene Set I and III (Figure 3). This suggests that BDE-209 dissolved in DMSO has a more pronounced effect than simply additive effects of BDE-209 and DMSO alone. Different solvents, for example, Tween 20 solution (Wang et al., 2011a), corn oil (Szabo et al., 2009), and ethanol (Ibhazehiebo et al., 2011), were used to dissolve PBDEs in studies of the effects on gene expression. This raises a caveat that one should set up an appropriate control to investigate the effect of a compound on gene expression.

### Carcinogenic potential of BDE-209

**Figure 4:**
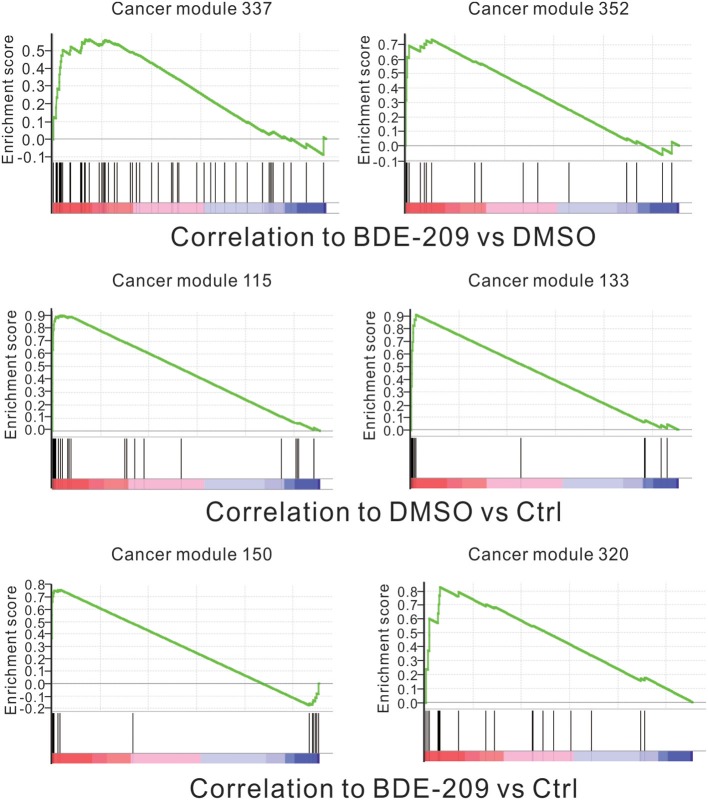
**Correlation between the enrichment of oncogenic signature genes and BDE-209 exposure**. Top panel: genes are ranked by the expression ratio of BDE-209 dissolved in DMSO sample over DMSO sample. The genes of cancer module 337 and 352 are positively correlated with BDE-209 exposure. Middle panel: genes are ranked by the expression ratio of DMSO sample over the negative control sample. The genes of cancer module 115 and 133 are positively correlated with DMSO treatment. Bottom panel: genes are ranked by the expression ratio of BDE-209 dissolved in DMSO sample over the negative control sample. The genes of cancer module 150 and 320 are positively correlated with BDE-209 exposure.

The standard 2-year rodent bioassays found dose-related increase in liver and thyroid tumors in both rats and mice with deca-BDE treatment (NTP, 1986). However, no statistically significant increases in tumors in an earlier bioassay in rats (Kociba et al., 1975). To fully test the carcinogenic potential effects of BDE-209 in HEK293T cells, we performed gene set enrichment analysis (GSEA) using all cancer-related gene sets curated by the molecular signatures database (MSigDB). The results show that the gene sets of two cancer modules (module 337 and 352) are positively correlated with BDE-209 treatment when compared with DMSO treatment (Figure 4). The gene sets of cancer modules were compiled from a large number of resources by mining published cancer-related microarray data (Segal et al., 2004). According to their functional annotation in MSigDB, the gene set of cancer module 337 play an important role in nucleotide metabolism. Their expression changes are involved in liver cancer, hematologic cancer and lung cancer. The gene set of cancer module 352 regulate nuclear pore complex. Their expression changes are correlated with hematologic cancer. The similar GSEA analysis found that gene sets of cancer modules 115 and 133 were enrichment in DMSO treatment when compared with the negative control (Figure 4). These gene sets have functions in prostate cancer, B lymphoma, and neurotumors. In contrast, the gene sets of cancer modules 150 and 320 are positively correlated with BDE-209 treatment when compared with the negative control (Figure 4). The gene sets of cancer modules 150 and 320 regulate translation elongation and M phase, respectively. The expression changes are related to various tumors, including prostate cancer, hematologic cancer, liver cancer, lung tumors, and B lymphoma. Taken together, BDE-209 exposure has carcinogenicity. Notably, there is no common gene sets among the gene sets positively correlated to each treatment. This implies that the carcinogenic potential of BDE-209 dissolved in DMSO is not simple additive potentials of BDE-209 and DMSO alone. It also suggests that one should take into account the dissolvent in the studies on the toxic effects of PBDEs.

## Discussion

PBDEs consist of more than 200 possible congeners. The commercial use of PBDEs as flame-retardant additives is actual in a mixture of congeners that are slowly released over time. PBDEs with different levels of bromination possess distinct properties. The fully brominated deca-BDE congeners are likely one of the least bioactive PBDEs because they are relatively poorly absorbed, rapidly removed, and not bioaccumulative (Hooper and McDonald, 2000). In contrast, PBDE congeners with lower levels of bromination, tri- to hexa-BDES, are almost fully absorbed, slowly degraded, and highly bioaccumulative when compared with deca-BDEs. Therefore, an important consideration is treatment time when one attempts to investigate the bona fide toxicity of deca-BDE before its elimination. It was reported that, when exposed to sunlight, deca-BDE could be converted to congeners with lower levels of bromination and become bioactive (Watanabe and Tatsukawa, 1987; Soderstrom et al., 2004). Consequently, studies on the toxic effect of the lower molecular weight congeners can more closely represent that of PBDEs in environment than deca-BDEs. Additionally, one single PBDE congener is usually used in studies on toxicity of PBDEs. However, the actual toxic effects of PBDE congeners leached to the environment from the commercial mixture are much more complex than that in bioassays.

As noted earlier, many bioassays on the toxic effects of PBDEs mainly focused on disruption of TH homeostasis and TH-induced biological processes. In this study, we used a not TH-related human cell HEK239T to investigate the toxic effects of PBDEs, and discovered that PBDE exposure could affect nucleosome organization. Additionally, BDE-209 has carcinogenic potential effects in HEK293T cells. Interestingly, a previous study showed that BDE-209 exposure can cause mouse sperm DNA damage in a dose dependent manner (Wang et al., 2011b). This finding suggests that PBDEs may exert different toxic effects on different type of cells. Collectively, TH has diverse toxic effects not limited to TH disruption as more studies are undertaken.

### Conflict of interest statement

The authors declare that the research was conducted in the absence of any commercial or financial relationships that could be construed as a potential conflict of interest.

## Supplementary material

Figure S1 **Frequency of ambiguous nucleotide (N) at each position of read sequences. RNA-seq reads have an extreme low portion of Ns for all three samples**.

Table S1 **RNA-seq read count of each sample**.

Table S2 **The differentially expressed genes between each two of the three samples**.
