# Supplementary material for: Toxic effects of decabromodiphenyl ether (BDE-209) on human embryonic kidney cells
Source: Front Genet. 2014 May 6;5:118. doi: 10.3389/fgene.2014.00118 (PMC4018524; doi:10.3389/fgene.2014.00118)
Supplement: Figure S1 — Frequency of ambiguous nucleotide (N) at each position of read sequences. RNA-seq reads have an extreme low portion of Ns for all three samples. [file DataSheet1.ZIP › Figure S1.pdf]

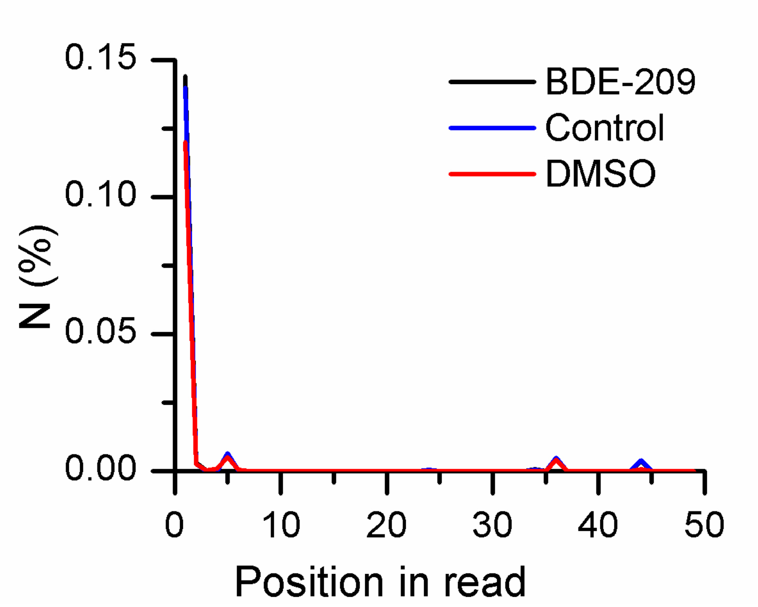

Figure S1. Frequency of ambiguous nucleotide (N) at each position of read sequences. RNA-seq reads have an extreme low portion of Ns for all three samples.
