# Supplementary material for: Toxic effects of decabromodiphenyl ether (BDE-209) on human embryonic kidney cells
Source: Front Genet. 2014 May 6;5:118. doi: 10.3389/fgene.2014.00118 (PMC4018524; doi:10.3389/fgene.2014.00118)
Supplement: Figure S1 — Frequency of ambiguous nucleotide (N) at each position of read sequences. RNA-seq reads have an extreme low portion of Ns for all three samples. [file DataSheet1.ZIP › Table S1.pdf]

Table S1. RNA-seq read count of each sample.

	**BDE-209**	**DMSO**	**Control**
Total clean reads	8,242,126	8,229,244	8,547,485
Mapped reads (%)	7,162,673 (86.90%)	7,127,425 (86.61)	7,425,293 (86.87%)
Uniquely mapped reads (%)	6,225,628 (75.53%)	6,194,267 (75.27)	6,457,368 (75.55%)
